# A Predictive Nomogram for Early Death of Metastatic Gastric Cancer: A Retrospective Study in the SEER Database and China

**DOI:** 10.7150/jca.46563

**Published:** 2020-07-14

**Authors:** Ying Zhu, Xiongfeng Fang, Lanqing Wang, Tao Zhang, Dandan Yu

**Affiliations:** 1 Cancer Center, Union Hospital, Tongji Medical College, Huazhong University of Science and Technology, Wuhan 430022, China.; 2 School of Electrical and Electronic Engineering, Huazhong University of Science and Technology, Wuhan 430074, China.

**Keywords:** Stomach neoplasm, Stage IV, SEER, Early death, Nomogram

## Abstract

**Background:** To identify associated risk factors and develop a predictive nomogram for the early death of metastatic gastric cancer patients.

**Methods:** A total of 4575 patients in the SEER cohort and 220 patients in the Chinese cohort diagnosed with metastatic gastric cancer in our Cancer Center were obtained. Univariate and multivariate logistic regression models were used to identify independent risk variables for early death. A predictive nomogram and a web-based probability calculator were developed and then validated by receiver operating characteristics (ROCs) curve and calibration plot in a Chinese cohort.

**Results:** Eight independent variables, including race, grade, surgery, chemotherapy, and metastases of bone, brain, liver, lung were recognized by using univariate and multivariate logistic regression models for identifying independent risk variables of early death about metastatic gastric cancer patients. By comprising these variables, a predictive nomogram and a web-based probability calculator were constructed in the SEER cohort. Then, it could be validated well in the Chinese cohort by receiver operating characteristics (ROCs) curve and calibration plot.

**Conclusion:** Using this nomogram model provided an insightful and applicable tool to distinguish the early death of metastatic gastric cancer patients.

## Introduction

Gastric cancer remains one of the most common malignancies all over the world, particularly in Asia 1-3. Most patients are diagnosed with advanced stages on account of unspecific symptoms 4, 5. There are still no curable treatments but only approved treatments like chemotherapy and immunotherapy for metastatic gastric cancer patients currently 6. Though targeted drugs can prolong the survival time of metastatic patients, the median is approximately 11-16 months 7. With the development of therapeutic approaches, metastatic gastric cancer patients have opportunities to survive better 8. However, early death (survival time ≤3 months) of metastatic gastric cancer patients remains unsolved problems 8. Therefore, it's necessary to identify risk factors of early death in gastric cancer patients. Such studies had been reported in colorectal cancer and ovarian cancer, but the researches in gastric cancer were rarely reported 9, 10.

This study aimed to recognize risk factors and establish a predictive nomogram for the early death of metastatic gastric cancer patients based on a large population cohort and a Chinese cohort.

## Materials and Methods

### Population

The SEER database, supported by the National Cancer Institute, constituted approximately 27.8% of the US population. SEER*Stat software (Version 8.3.6) was used to extract clinical information. We applied the primary site codes C16.0-C16.9 for gastric and the International Classification of Diseases for Oncology, Third Edition (ICD-O-3) histologic codes 8140/3 (Adenocarcinoma, NOS), 8211/3 (Tubular adenocarcinoma), 8260/3 (Papillary adenocarcinoma), 8480/3 (Mucinous adenocarcinoma), 8490/3 (Signet ring cell carcinoma) for adenocarcinoma. The information for metastatic sites of the bone (SEER Combined Mets at DX-bone), brain (SEER Combined Mets at DX-brain), liver (SEER Combined Mets at DX-liver), and lung (SEER Combined Mets at DX-lung) were collected since 2010, thus metastatic gastric cancer patients diagnosed stage IV and from 2010 to 2015 were included. Patients of metastatic gastric cancer without histological confirmation, only one primary malignancy, survival months, race, marital status, grade, and cause of death were excluded. Surgery was not recommended in metastatic gastric cancer; therefore T stage and N stage without specific information were accepted. Demographic and clinical characteristics were withdrawn in the SEER database as follows: age, sex, race, marital status, primary site, histology, grade, bone metastases, brain metastases, liver metastases, lung metastases, T stage, N stage, surgery, radiation, chemotherapy, cause of death, survival months.

The clinical data for metastatic gastric cancer patients initially diagnosed at Cancer Center, Union Hospital between January 2011 and September 2018 were collected retrospectively. The last follow-up was in January 2019. The inclusion and exclusion criteria mentioned above for the SEER cohort were also applied to the Chinese cohort. Surgery was not observed in the Chinese cohort and marital status was not recorded as well. T stage and N stage were mainly based on CT or endoscopic ultrasonography. Besides, smoking and drinking history were recorded. Death less than 3 months since the first diagnosis was regarded as early death in patients with malignant tumors.

This retrospective study of the Chinese cohort was approved by the ethics committee of Tongji Medical College, Huazhong University of Science and Technology in accordance with the ethical standards prescribed by the Helsinki Declaration.

### Nomogram Development and Statistical analysis

Categorical data were described by the number and the percentage (N, %). Univariate and multivariate logistic regression models were utilized to identify variables that significantly associated with the early death of metastatic gastric cancer in the SEER sets. Then the predictive nomogram based on independent factors was constructed for the early death of metastatic gastric cancer by the SEER cohort. To evaluate the calibration and discrimination of the nomogram, an external independent Chinese patient validation cohort was used. For calibration, the nomogram predicted probabilities were contrasted with the actual probabilities by bootstrapping with 1000 resamples. The receiver operating characteristic (ROC) curve was used to judge discrimination. The higher the area under the curve (AUC) was, the better the accuracy would be. The “DynNom” and “shiny” packages were used to construct a web-based probability calculator, which could dynamically predict the probability of early death.

All analyses were performed using the R software (version 3.6.2). A two-tailed *P* value less than 0.01 was considered statistically significant.

## Results

### Demographic and clinical characteristics

There were 4575 patients in the SEER database and 220 patients in our cancer center enrolled in this study. The demographic and clinical characteristics of metastatic gastric cancer were listed in Table 1.

For the SEER cohort, most patients (82.4%) were between 40 to 79 years old. 65.3% of the patients are male and 61.3% are married. The main primary sites were gastric antrum and cardiac. The most common metastatic sites were liver, accounting for approximately 43.2% of liver metastases. Only 10.3% of patients performed surgery and 19.6% of patients performed radiation. There were nearly 67.6% of patients that received chemotherapy. The majority of metastatic gastric cancer patients died of the primary disease, while few of them died of other causes such as heart disease, cerebrovascular disease, septicemia, and so on.

In the Chinese cohort, the majority of patients (91.9%) were between 40 to 79 years old. The proportion of male patients was nearly equal to female patients. Approximately 20.9% and 20.0% of patients had a smoking history and drinking history, respectively. Metastatic gastric cancer patients constituted 32.3% of the liver metastases. Most patients (72.3%) received chemotherapy.

### Identifying independent factors for early death

By applying univariate and multivariate logistic regression in the SEER cohort, the risk variables for the early death of metastatic gastric cancer were analyzed (Table 2). Univariate logistic models showed that age, marital status, primary site, grade, metastases of bone, brain metastases, metastases of liver, lung metastases, T stage, N stage, surgery, radiation, and chemotherapy were associated with early death. Multivariate analyses proved eight independent risk factors including race, grade, surgery, chemotherapy, and metastases of bone, brain, liver, lung, which were significantly related to the early death of metastatic gastric cancer.

### Nomogram construction

**Figure 1:**
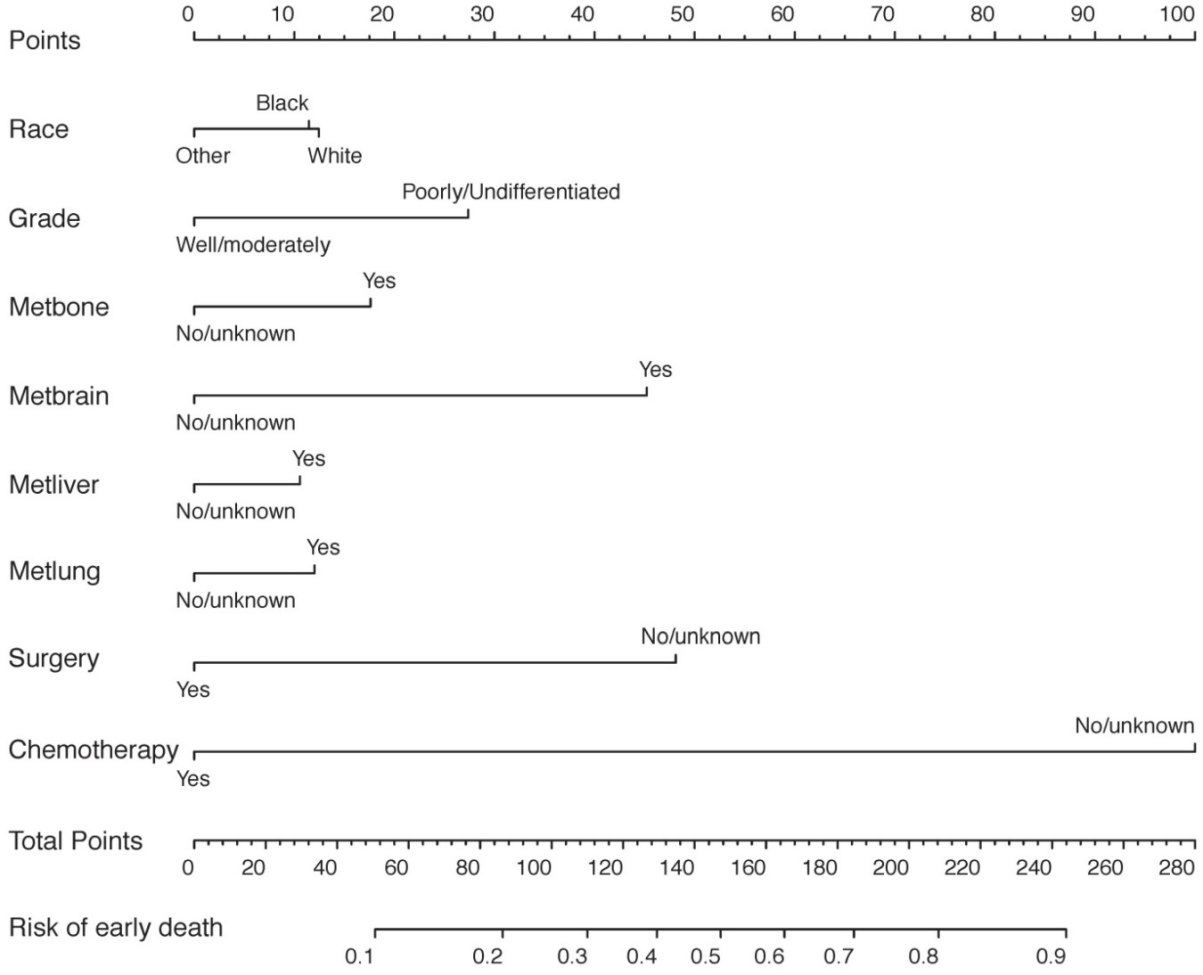
The predictive nomogram for the early death of metastatic gastric cancer patients in the SEER database.

The predictive nomogram relying on identified risk factors from multivariate logistic regression models in the SEER cohort was constructed in Figure 1. By calculating every point of variables, the total number of points could be associated with the probability of early death of metastatic gastric cancer.

### Nomogram validation

**Figure 2:**
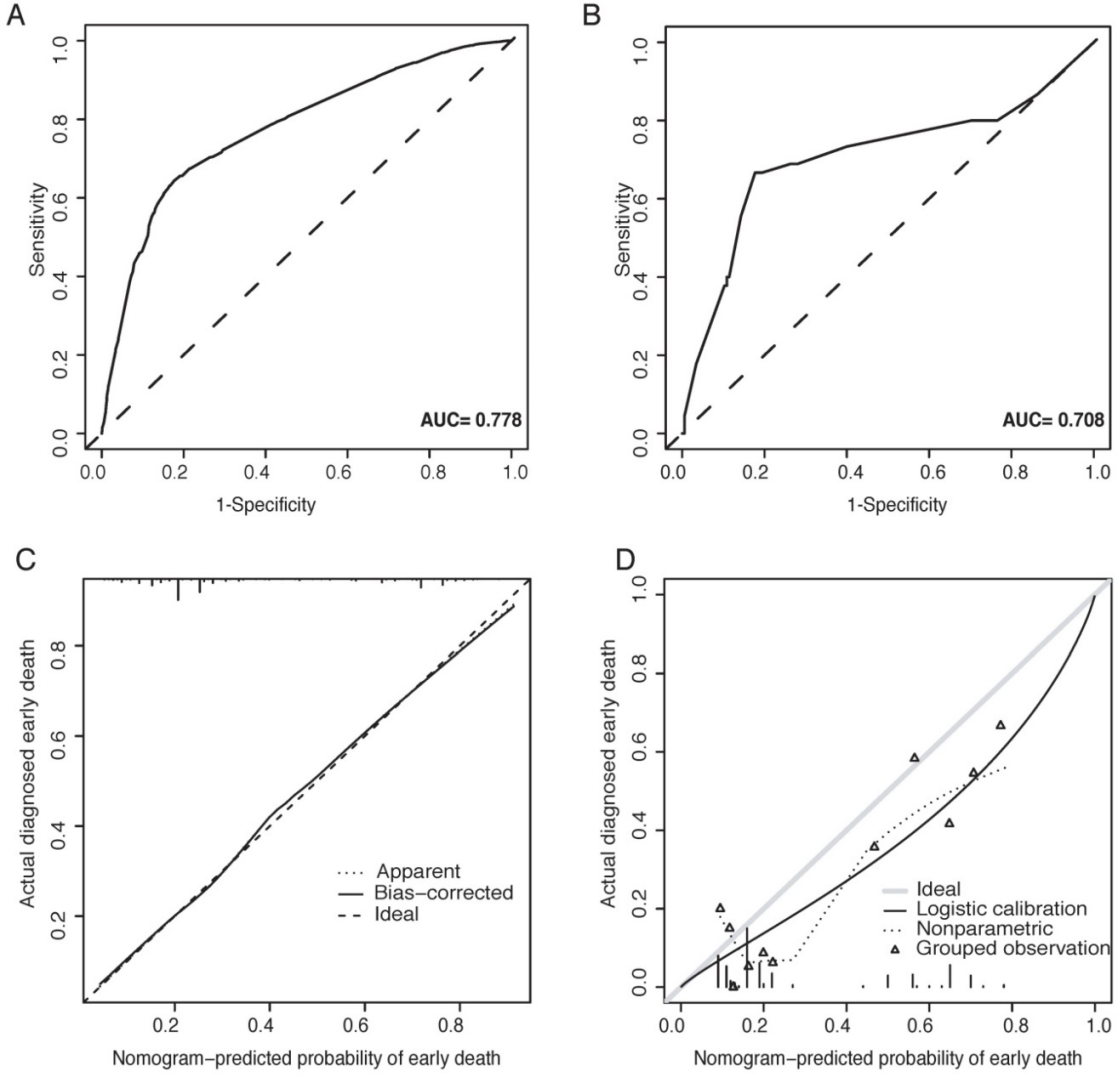
ROC curves and calibration plots for the nomogram. (A) The ROC curve for the nomogram in the SEER cohort; (B) the ROC curve for the nomogram in the Chinese cohort; (C) the calibration plots for the nomogram in the SEER cohort; (D) the calibration plots for the nomogram in the Chinese cohort.

The predictive nomogram was validated both in the SEER cohort and the Chinese cohort. For the Chinese cohort, nomogram predicted probabilities of early death were computed according to the nomogram based on the SEER cohort. The calibration plots performed well both in the training and validation cohort (Figure 2C-D). Besides, applicable AUC was shown in both sets (Figure 2A-B).

### Web-based probability calculator

**Figure 3:**
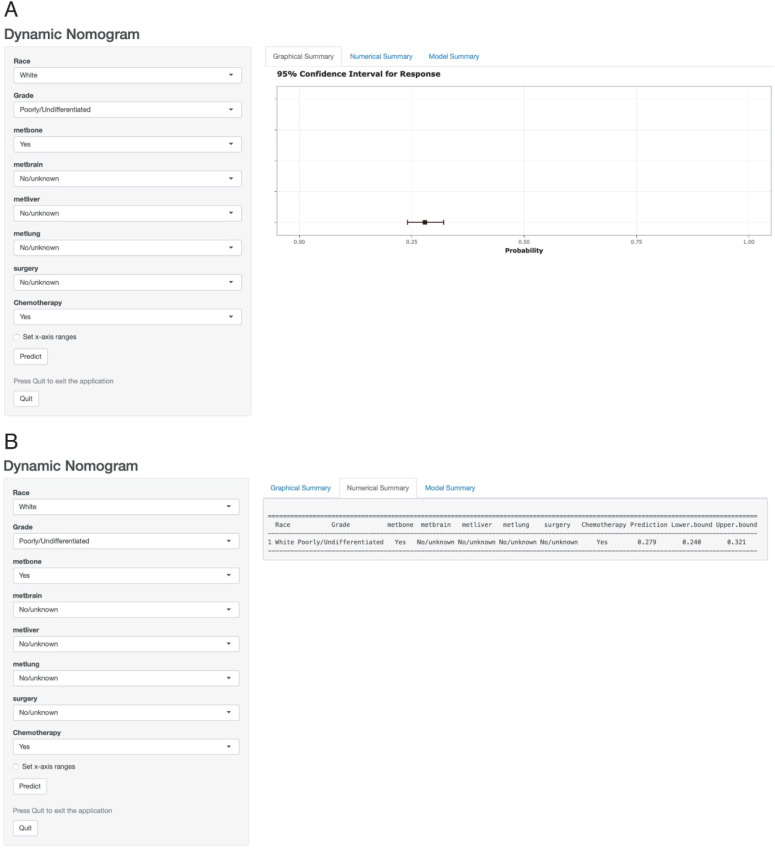
A web-based probability calculator. White patients with a poorly or differentiated grade, bone metastasis, chemotherapy and without brain metastasis, liver metastasis, lung metastasis, surgery showed in the web probability calculator. (A) The graphical summary showed a rough range. (B) Numerical Summary showed the early death probability and its 95% confidence interval.

According to the above results, a dynamic web-based probability calculator (https:/wandertheworld.shinyapps.io/DynNomapp/) was constructed to predict the early death of metastatic gastric cancer patients on the basis of the previous nomogram (Figure 3A). It's very convenient to individually predict the early death probability of patients based on their clinical characteristics. For instance, the early death probability of metastatic gastric cancer was approximately 27.9% (95% *CI*: 24.0-32.1%) for white patients with a poorly or differentiated grade, bone metastasis, chemotherapy, and without brain metastasis, liver metastasis, lung metastasis, surgery (Figure 3B).

## Discussion

The incidence and morbidity of gastric cancer remained high worldwide 11. Thanks to the development of a number of treatments including targeted drugs, immunotherapy, and radiation, the median survival time had been prolonged all the time 12-16. However, one-third and one-fifth of early death which was defined as death came up within 3 months occurred in the SEER cohort and the Chinese cohort of this study, respectively. Researchers concentrated deeply on associated independent variables of overall survival, cancer-specific survival, and noncancer-specific survival in gastric cancer 17-19. And nomograms had been widely developed and used for predicting long-term survival of cancers 20-24. To the best of our knowledge, this was the first study to identify the associated risk factors and structure an available nomogram for recognizing the early death of metastatic gastric cancer patients.

For the SEER cohort, multivariate analyses identified eight independent risk factors, including race, grade, surgery, chemotherapy, and metastases of bone, brain, liver, lung. Some previous studies had reported that grade, surgery, chemotherapy, and distant metastasis were significantly related to overall survival 25, 26. This study firstly confirmed that these risk factors were related to early death. Since some studies showed that metastatic gastric cancer patients performing surgical resection had a relatively poor prognosis, the surgery for metastatic gastric cancer patients remained controversial 27. Furthermore, for those stage IV gastric cancer patients with synchronous liver metastasis, surgery had been reconsidered with the improvement of longer overall survival time 28, 29.

There were apparent differences between the SEER cohort and the Chinese cohort. A lot of factors might result in the differences, including incidence, insurance, economic conditions, treatment strategy, religious beliefs, and so on.

A convenient nomogram containing identified independent factors was constructed for predicting early death of metastatic gastric cancer patients. Once they were distinguished, closer attention and better therapeutic strategies such as clinical trials might be given by oncologists.

There were several limitations to this study. First, risk factors associated with early death, including peritoneal metastasis, numbers of metastatic sites, performance status, and background diseases were absent. Second, the excluded missing data might result in selection bias. Third, though a Chinese cohort was used to validate the nomogram, a large prospective study was requisite.

All in all, a comprehensive nomogram integrating identified risk factors and a web-based probability calculator based on the nomogram were constructed to distinguish the early death of metastatic gastric cancer patients. The nomogram might help oncologists to make better therapeutic strategies such as clinical trials, and hospice management.

## Figures and Tables

**Table 1:** Demographic and clinical characteristics in the SEER database and Chinese cohort

Characteristic	SEER cohort					Chinese cohort				
	Total death n=4575 (%)	Died 1-90 days, n=1647 (%)	Died 91-180 days, n=863 (%)	Died 181-360 days, n=1075 (%)	Died >1-year, n=990 (%)	Total death n=220 (%)	Died 1-90 days, n=45 (%)	Died 91-180 days, n=48 (%)	Died 181-360 days, n=70 (%)	Died >1-year, n=57 (%)
**Age (years)**										
<40	261 (5.7)	72 (4.4)	56 (6.5)	79 (7.3)	54 (5.5)	17 (7.7)	5 (11.1)	1 (2.1)	3 (4.3)	8 (14.0)
40-59	1581 (34.6)	463 (28.1)	313 (36.3)	411 (38.2)	394 (39.8)	100 (45.5)	17 (37.8)	27 (56.3)	35 (50.0)	21 (36.8)
60-79	2185 (47.8)	799 (48.5)	387 (44.8)	517 (48.1)	482 (48.7)	102 (46.4)	23 (51.1)	20 (41.7)	31 (44.3)	28 (49.1)
80+	548 (12.0)	313 (19.0)	107 (12.4)	68 (6.3)	60 (6.1)	1 (0.5)	0 (0.0)	0 (0.0)	1 (1.4)	0 (0.0)
**Sex**										
Male	2986 (65.3)	1070 (65.0)	560 (64.9)	700 (65.1)	656 (66.3)	113 (51.4)	22 (48.9)	20 (41.7)	38 (54.3)	33 (57.9)
Female	1589 (34.7)	577 (35.0)	303 (35.1)	375 (34.9)	334 (33.7)	107 (48.6)	23 (51.1)	28 (58.3)	32 (45.7)	24 (42.1)
**Race**										
White	3384 (74.0)	1229 (74.6)	622 (72.1)	807 (75.1)	726 (73.3)	0 (0.0)	0 (0.0)	0 (0.0)	0 (0.0)	0 (0.0)
Black	558 (12.2)	214 (13.0)	98 (11.4)	122 (11.3)	124 (12.5)	0 (0.0)	0 (0.0)	0 (0.0)	0 (0.0)	0 (0.0)
Others	633 (13.8)	204 (12.4)	143 (16.6)	146 (13.6)	140 (14.1)	220 (100.0)	45 (100.0)	48 (100.0)	70 (100.0)	57 (100.0)
**Marital status**										
Married	2806 (61.3)	896 (54.4)	538 (62.3)	715 (66.5)	657 (66.4)	NA	NA	NA	NA	NA
Separated/Divorced	489 (10.7)	188 (11.4)	88 (10.2)	108 (10.0)	105 (10.6)	NA	NA	NA	NA	NA
Widowed	472 (10.3)	242 (14.7)	89 (10.3)	76 (7.1)	65 (6.6)	NA	NA	NA	NA	NA
Single	808 (17.7)	321 (19.5)	148 (17.1)	176 (16.4)	163 (16.5)	NA	NA	NA	NA	NA
**Smoking history**										
Yes	NA	NA	NA	NA	NA	46 (20.9)	11 (24.4)	10 (20.8)	13 (18.6)	12 (21.1)
No	NA	NA	NA	NA	NA	174 (79.1)	34 (75.6)	38 (79.2)	57 (81.4)	45 (78.9)
**Drinking history**										
Yes	NA	NA	NA	NA	NA	44 (20.0)	8 (17.8)	10 (20.8)	16 (22.9)	10 (17.5)
No	NA	NA	NA	NA	NA	176 (80.0)	37 (82.2)	38 (79.2)	54 (77.1)	47 (82.5)
**Primary site**										
Cardiac/fundus	1907 (41.7)	626 (38.0)	353 (40.9)	469 (43.6)	459 (46.4)	52 (23.6)	6 (13.3)	10 (20.8)	23 (32.9)	13 (22.8)
Body	451 (9.9)	158 (9.6)	98 (11.4)	110 (10.2)	85 (8.6)	55 (25.0)	17 (37.8)	12 (25.0)	15 (21.4)	11 (19.3)
Antrum/pylorus	690 (15.1)	269 (16.3)	113 (13.1)	156 (14.5)	154 (15.6)	60 (27.3)	14 (31.1)	8 (16.7)	17 (24.3)	21 (36.8)
Lesser/greater curvature	846 (18.5)	140 (8.5)	79 (9.2)	100 (9.3)	82 (8.3)	2 (0.9)	1 (2.2)	1 (2.1)	0 (0.0)	0 (0.0)
Other	681 (14.9)	454 (27.6)	220 (25.5)	243 (22.6)	210 (21.1)	51 (23.2)	7 (15.6)	17 (35.4)	15 (21.4)	12 (21.1)
**Histology**										
Adenocarcinoma	3358 (73.4)	1232 (74.8)	626 (72.5)	767 (71.3)	733 (74.0)	182 (82.7)	37 (82.2)	41 (85.4)	59 (84.3)	45 (78.9)
Signet ring cell carcinoma	1217 (26.6)	415 (25.2)	237 (27.5)	308 (28.7)	257 (26.0)	38 (17.3)	8 (17.8)	7 (14.6)	11 (15.7)	12 (21.1)
**Grade**										
Well/moderately	1101 (24.1)	344 (20.9)	194 (22.5)	276 (25.7)	287 (29.0)	182 (82.7)	37 (82.2)	41 (85.4)	59 (84.3)	45 (78.9)
Poorly /undifferentiated	3474 (75.9)	1303 (79.1)	669 (77.5)	799 (74.3)	703 (71.0)	38 (17.3)	8 (17.8)	7 (14.6)	11 (15.7)	12 (21.1)
**Bone metastases**										
Yes	607 (13.3)	254 (15.4)	122 (14.1)	146 (13.6)	85 (8.6)	24 (10.9)	4 (8.9)	8 (16.7)	6 (8.6)	6 (10.5)
No/unknown	3968 (86.7)	1393 (84.6)	741 (85.9)	929 (86.4)	905 (91.4)	196 (89.1)	41 (91.1)	40 (83.3)	64 (91.4)	51 (89.5)
**Brain metastases**										
Yes	95 (2.1)	55 (3.3)	17 (2.0)	9 (0.8)	14 (1.4)	2 (0.9)	0 (0.0)	1 (2.1)	1 (1.4)	0 (0.0)
No/unknown	4480 (97.9)	1592 (96.7)	846 (98.0)	1066 (99.2)	976 (98.6)	218 (99.1)	45 (100.0)	47 (97.9)	69 (98.6)	57 (100.0)
**Liver metastases**										
Yes	1975 (43.2)	764 (46.4)	366 (42.4)	453 (42.1)	392 (39.6)	71 (32.3)	18 (40.0)	15 (31.3)	20 (28.6)	18 (31.6)
No/unknown	2600 (56.8)	883 (53.6)	497 (57.6)	622 (57.9)	598 (60.4)	149 (67.7)	27 (60.0)	33 (68.8)	50 (71.4)	39 (68.4)
**Lung metastases**										
Yes	656 (14.3)	275 (16.7)	127 (14.7)	145 (13.5)	109 (11.0)	14 (6.4)	1 (2.2)	4 (8.3)	5 (7.1)	4 (7.0)
No/unknown	3919 (85.7)	1372 (83.3)	736 (85.3)	930 (86.5)	881 (89.0)	206 (93.6)	44 (97.8)	44 (81.7)	65 (92.9)	53 (93.0)
**T stage**										
T1	822 (18.0)	302 (18.3)	161 (18.7)	190 (17.7)	169 (17.1)	1 (0.5)	1 (2.2)	0 (0.0)	0 (0.0)	0 (0.0)
T2	200 (4.4)	51 (3.1)	35 (4.1)	45 (4.2)	69 (7.0)	3 (1.4)	0 (0.0)	1 (2.1)	0 (0.0)	2 (3.5)
T3	742 (16.2)	205 (12.4)	141 (16.3)	191 (17.8)	205 (20.7)	24 (10.9)	4 (8.9)	2 (4.2)	10 (14.3)	8 (14.0)
T4	1004 (21.9)	354 (21.5)	192 (22.2)	249 (23.2)	209 (21.1)	49 (22.3)	5 (11.1)	6 (12.5)	13 (18.6)	25 (43.9)
Unknown	1807 (39.5)	735 (44.6)	334 (38.7)	400 (37.2)	338 (34.1)	143 (65.0)	35 (77.8)	39 (81.3)	47 (67.1)	22 (38.6)
**N stage**										
N0	1542 (33.7)	613 (37.2)	294 (34.1)	321 (29.9)	314 (31.7)	4 (1.8)	1 (2.2)	1 (2.1)	0 (0.0)	2 (3.5)
N1	1708 (37.3)	545 (33.1)	333 (38.6)	449 (41.8)	381 (38.5)	5 (2.3)	0 (0.0)	0 (0.0)	2 (2.9)	3 (5.3)
N2	301 (6.6)	72 (4.4)	51 (5.9)	75 (7.0)	103 (10.4)	23 (10.5)	3 (6.7)	0 (0.0)	12 (17.1)	8 (14.0)
N3	305 (6.7)	79 (4.8)	60 (7.0)	79 (7.3)	87 (8.8)	32 (14.5)	2 (4.4)	7 (14.6)	6 (8.6)	17 (29.8)
Unknown	719 (15.7)	338 (20.5)	125 (14.5)	151 (14.0)	105 (10.6)	156 (70.9)	39 (86.7)	40 (83.3)	50 (71.4)	27 (47.4)
**Surgery**										
Yes	470 (10.3)	99 (6.0)	68 (7.9)	125 (11.6)	178 (18.0)	0 (0.0)	0 (0.0)	0 (0.0)	0 (0.0)	0 (0.0)
No/unknown	4105 (89.7)	1548 (94.0)	795 (92.1)	950 (88.4)	812 (82.0)	220 (100.0)	45 (100.0)	48 (100.0)	70 (100.0)	57 (100.0)
**Radiation**										
Yes	896 (19.6)	289 (17.5)	167 (19.4)	226 (21.0)	214 (21.6)	14 (6.4)	0 (0.0)	1 (2.1)	7 (10.0)	6 (10.5)
No/unknown	3679 (80.4)	1358 (82.5)	696 (80.6)	849 (79.0)	776 (78.4)	206 (93.6)	45 (100.0)	47 (97.9)	63 (90.0)	51 (89.5)
**Chemotherapy**										
Yes	3096 (67.6)	639 (38.8)	640 (74.2)	923 (85.9)	892 (90.1)	159 (72.3)	15 (33.3)	41 (85.4)	56 (80.0)	47 (82.5)
No/unknown	1481 (32.4)	1008 (61.2)	223 (25.8)	152 (14.1)	98 (9.9)	61 (27.7)	30 (66.7)	7 (14.6)	14 (20.0)	10 (17.5)
**Cause of death**										
Gastric cancer	4434 (96.9)	1588 (96.4)	842 (97.6)	1044 (97.1)	960 (97.0)	NA	NA	NA	NA	NA
Other causes	141 (3.1)	59 (3.6)	21 (2.4)	31 (2.9)	30 (3.0)	NA	NA	NA	NA	NA

Abbreviations: not available, NA.

**Table 2:** Univariate and multivariate logistic regression for analyzing the risk factors for early death in the SEER database

Variables	Univariate			Multivariate		
	OR	95%CI	*p* value	OR	95%CI	*p* value
**Age (years)**						
<40	Ref			Ref		
40-59	1.087	0.815-1.463	0.575	0.836	0.603-1.168	0.286
60-79	1.513	1.143-2.023	**0.004**	1.106	0.799-1.545	0.548
80+	3.496	2.549-4.836	**<0.001**	1.458	0.993-2.152	0.056
**Sex**						
Male	Ref			Ref		
Female	1.021	0.899-1.159	0.748	0.828	0.704-0.973	0.022
**Race**						
White	Ref			Ref		
Black	1.091	0.906-1.311	0.356	0.910	0.727-1.137	0.410
Other	0.834	0.695-0.998	0.049	0.706	0.569-0.874	**0.001**
**Marital status**						
Married	Ref			Ref		
Separated/Divorced	0.049	1.090-1.623	**0.005**	1.305	1.034-1.642	0.024
Widowed	2.243	1.842-2.732	**<0.001**	1.241	0.963-1.598	0.094
Single	1.405	1.195-1.651	**<0.001**	1.273	1.047-1.547	0.015
**Primary site**						
Cardiac/fundus	Ref			Ref		
Body	1.103	0.888-1.367	0.371	1.201	0.921-1.561	0.174
Antrum/pylorus	1.308	1.091-1.565	**0.004**	1.305	1.036-1.644	0.023
Lesser& greater curvature	1.184	0.999-1.401	0.051	1.314	1.064-1.620	0.011
Other	1.464	1.222-1.752	**<0.001**	1.148	0.919-1.435	0.223
**Histology**						
Adenocarcinoma	Ref			Ref		
Signet ring cell carcinoma	0.893	0.778-1.024	0.107	0.999	0.831-1.201	0.996
**Grade**						
Well/moderately	Ref			Ref		
Poorly /undifferentiated	1.321	1.144-1.528	**<0.001**	1.904	1.588-2.287	**<0.001**
**Bone metastases**						
Yes	Ref			Ref		
No/unknown	0.752	0.632-0.895	**0.001**	0.64	0.521-0.786	**<0.001**
**Brain metastases**						
Yes	Ref			Ref		
No/unknown	0.401	0.264-0.603	**<0.001**	0.328	0.202-0.529	**<0.001**
**Liver metastases**						
Yes	Ref			Ref		
No/unknown	0.815	0.722-0.921	**0.001**	0.798	0.682-0.934	**0.005**
**Lung metastases**						
Yes	Ref			Ref		
No/unknown	0.746	0.631-0.884	**0.001**	0.749	0.614-0.915	**0.004**
**T stage**						
T1	Ref			Ref		
T2	0.589	0.413-0.830	**0.003**	0.591	0.391-0.883	0.011
T3	0.657	0.530-0.814	**<0.001**	0.928	0.719-1.197	0.566
T4	0.938	0.774-1.136	0.512	1.121	0.887-1.418	0.341
Unknown	1.181	0.996-1.400	0.056	1.051	0.858-1.289	0.630
**N stage**						
N0	Ref			Ref		
N1	0.710	0.615-0.820	**<0.001**	0.821	0.692-0.973	0.023
N2	0.476	0.357-0.630	**<0.001**	0.703	0.500-0.982	0.041
N3	0.530	0.400-0.695	**<0.001**	0.783	0.553-1.102	0.164
Unknown	1.344	1.125-1.607	**0.001**	1.245	1.005-1.542	0.045
**Surgery**						
Yes	Ref			Ref		
No/unknown	2.269	1.809-2.870	**<0.001**	2.697	1.993-3.675	**<0.001**
**Radiation**						
Yes	Ref			Ref		
No/unknown	1.229	1.053-1.437	**0.009**	0.985	0.815-1.193	0.879
**Chemotherapy**						
Yes	Ref			Ref		
No/unknown	8.187	7.125-9.422	**<0.001**	8.53	7.308-9.978	**<0.001**

## References

[1] Thrift AP, El-Serag HB (2020). Burden of Gastric Cancer. Clin Gastroenterol Hepatol.

[2] Chen W, Zheng R, Baade PD, Zhang S, Zeng H, Bray F (2016). Cancer statistics in China, 2015. CA: a cancer journal for clinicians.

[3] Rahman R, Asombang AW, Ibdah JA (2014). Characteristics of gastric cancer in Asia. World J Gastroenterol.

[4] Humphrys E, Walter FM, Rubin G, Emery JD, Johnson M, Richards A (2020). Patient symptom experience prior to a diagnosis of oesophageal or gastric cancer: a multi-methods study. BJGP Open.

[5] Monti M, Massa I, Foca F, Morgagni P, Framarini M, Passardi A (2020). Retrospective analysis of gastric cancer management in a real-world setting: a single-institution experience. Tumori.

[6] Chen L-T, Satoh T, Ryu M-H, Chao Y, Kato K, Chung HC (2020). A phase 3 study of nivolumab in previously treated advanced gastric or gastroesophageal junction cancer (ATTRACTION-2): 2-year update data. Gastric cancer: official journal of the International Gastric Cancer Association and the Japanese Gastric Cancer Association.

[7] Bang Y-J, Van Cutsem E, Feyereislova A, Chung HC, Shen L, Sawaki A (2010). Trastuzumab in combination with chemotherapy versus chemotherapy alone for treatment of HER2-positive advanced gastric or gastro-oesophageal junction cancer (ToGA): a phase 3, open-label, randomised controlled trial. Lancet (London, England).

[8] Lee J, Kim ST, Kim K, Lee H, Kozarewa I, Mortimer PGS (2019). Tumor Genomic Profiling Guides Patients with Metastatic Gastric Cancer to Targeted Treatment: The VIKTORY Umbrella Trial. Cancer Discov.

[9] Wang X, Mao M, Xu G, Lin F, Sun P, Baklaushev VP (2019). The incidence, associated factors, and predictive nomogram for early death in stage IV colorectal cancer. Int J Colorectal Dis.

[10] Urban RR, He H, Alfonso R, Hardesty MM, Gray HJ, Goff BA (2016). Ovarian cancer outcomes: Predictors of early death. Gynecol Oncol.

[11] Siegel RL, Miller KD, Jemal A (2020). Cancer statistics, 2020. CA: a cancer journal for clinicians.

[12] Karrit S, Belaid I, Ezzairi F, El Ghali A, Bedioui A, Bouzid N (2018). Management, outcome and prognostic factors of metastatic gastric cancer. Annals of oncology: official journal of the European Society for Medical Oncology.

[13] Sasaki A, Nakamura Y, Togashi Y, Kuno H, Hojo H, Kageyama S (2020). Enhanced tumor response to radiotherapy after PD-1 blockade in metastatic gastric cancer. Gastric cancer: official journal of the International Gastric Cancer Association and the Japanese Gastric Cancer Association.

[14] Ishigami H, Fujiwara Y, Fukushima R, Nashimoto A, Yabusaki H, Imano M (2018). Phase III Trial Comparing Intraperitoneal and Intravenous Paclitaxel Plus S-1 Versus Cisplatin Plus S-1 in Patients With Gastric Cancer With Peritoneal Metastasis: PHOENIX-GC Trial. Journal of clinical oncology: official journal of the American Society of Clinical Oncology.

[15] Guimbaud R, Louvet C, Ries P, Ychou M, Maillard E, André T (2014). Prospective, randomized, multicenter, phase III study of fluorouracil, leucovorin, and irinotecan versus epirubicin, cisplatin, and capecitabine in advanced gastric adenocarcinoma: a French intergroup (Fédération Francophone de Cancérologie Digestive, Fédération Nationale des Centres de Lutte Contre le Cancer, and Groupe Coopérateur Multidisciplinaire en Oncologie) study. Journal of clinical oncology: official journal of the American Society of Clinical Oncology.

[16] Li J, Qin S, Xu J, Xiong J, Wu C, Bai Y (2016). Randomized, Double-Blind, Placebo-Controlled Phase III Trial of Apatinib in Patients With Chemotherapy-Refractory Advanced or Metastatic Adenocarcinoma of the Stomach or Gastroesophageal Junction. Journal of clinical oncology: official journal of the American Society of Clinical Oncology.

[17] Park HS, Kwon WS, Park S, Jo E, Lim SJ, Lee C-K (2019). Comprehensive immune profiling and immune-monitoring using body fluid of patients with metastatic gastric cancer. J Immunother Cancer.

[18] Liu M, Wang R, Sun X, Liu Y, Wang Z, Yan J (2020). Prognostic significance of PD-L1 expression on cell-surface vimentin-positive circulating tumor cells in gastric cancer patients. Mol Oncol.

[19] Oh C-M, Lee D, Kong H-J, Lee S, Won Y-J, Jung K-W (2020). Causes of death among cancer patients in the era of cancer survivorship in Korea: Attention to the suicide and cardiovascular mortality. Cancer Med.

[20] Liu J, Geng Q, Liu Z, Chen S, Guo J, Kong P (2016). Development and external validation of a prognostic nomogram for gastric cancer using the national cancer registry. Oncotarget.

[21] Chen D, Jiang B, Xing J, Liu M, Cui M, Liu Y (2013). Validation of the memorial Sloan-Kettering Cancer Center nomogram to predict disease-specific survival after R0 resection in a Chinese gastric cancer population. PLoS ONE.

[22] Zhao R, Jia T, Qiao B, Liang J, Qu S, Zhu L (2019). Nomogram predicting long-term overall survival and cancer-specific survival of lip carcinoma patients based on the SEER database: A retrospective case-control study. Medicine.

[23] Cao L-L, Lu J, Lin J-X, Zheng C-H, Li P, Xie J-W (2018). Incidence and survival trends for gastric neuroendocrine neoplasms: An analysis of 3523 patients in the SEER database. European journal of surgical oncology: the journal of the European Society of Surgical Oncology and the British Association of Surgical Oncology.

[24] Chen Z, Lin R-M, Bai Y-K, Zhang Y (2019). Establishment and Verification of Prognostic Nomograms for Patients with Gastrointestinal Stromal Tumors: A SEER-Based Study. Biomed Res Int.

[25] Kawahara K, Makino H, Kametaka H, Hoshino I, Fukada T, Seike K (2020). Outcomes of surgical resection for gastric cancer liver metastases: a retrospective analysis. World J Surg Oncol.

[26] Mikami J, Kimura Y, Makari Y, Fujita J, Kishimoto T, Sawada G (2017). Clinical outcomes and prognostic factors for gastric cancer patients with bone metastasis. World J Surg Oncol.

[27] Fujitani K, Yang H-K, Mizusawa J, Kim Y-W, Terashima M, Han S-U (2016). Gastrectomy plus chemotherapy versus chemotherapy alone for advanced gastric cancer with a single non-curable factor (REGATTA): a phase 3, randomised controlled trial. The Lancet Oncology.

[28] Kodera Y, Fujitani K, Fukushima N, Ito S, Muro K, Ohashi N (2014). Surgical resection of hepatic metastasis from gastric cancer: a review and new recommendation in the Japanese gastric cancer treatment guidelines. Gastric cancer: official journal of the International Gastric Cancer Association and the Japanese Gastric Cancer Association.

[29] Picado O, Dygert L, Macedo FI, Franceschi D, Sleeman D, Livingstone AS (2018). The Role of Surgical Resection for Stage IV Gastric Cancer With Synchronous Hepatic Metastasis. The Journal of surgical research.

